# Surgical management of a midline submental neck mass in Zimbabwe: A case report

**DOI:** 10.4102/jcmsa.v2i1.20

**Published:** 2024-05-07

**Authors:** Dontre’ Douse, Katerina Green, Chengetai Dziwa, Munyaradzi Katiro, Tafadzwa Nyamurowa, Farai Ethel Chikomba, Titus Dzongodza, Joshua Wiedermann

**Affiliations:** 1 Department of Otolaryngology-Head and Neck Surgery, Mayo Clinic, Rochester, United States of America; 2 Department of Otolaryngology-Head and Neck Surgery, Mayo Clinic, Jacksonville, United States of America; 3 Department of Otolaryngology-Head and Neck Surgery, University of Zimbabwe, Harare, Zimbabwe

**Keywords:** dermoid, midline neck mass, neck mass, pediatric neck mass, congenital neck mass

## Abstract

**Introduction:**

Differential diagnoses of a pediatric neck mass are extensive, ranging across benign, malignant, congenital and infectious causes. Limited access to imaging in low-resource settings increases the complexity of narrowing this list to appropriately diagnose and treat neck masses. This occasionally allows neck masses to grow unchecked, increasing the morbidity of their presence and eventual excision. The goal of this report is to describe nuances in the diagnosis and treatment of pediatric neck masses in resource-limited settings through a case report of a neck mass in a pediatric patient.

**Patient presentation:**

A 10-year-old girl in Harare, Zimbabwe, presented for surgical management of a midline neck mass that appeared 4 years prior to presentation for definitive treatment. A myriad of barriers delayed her care, allowing the mass to grow into her floor of mouth, displacing her tongue and leading to speech and feeding difficulties.

**Management and outcome:**

At presentation to the tertiary care facility, she received a pre-operative ultrasound showing a well-circumscribed, echogenic mass. Her neck mass was surgically excised through a transoral approach. Permanent pathology was consistent with a dermoid cyst.

**Conclusion:**

At 1-month postoperative follow-up, the patient had experienced no complications and had a complete resolution of her functional symptoms and appearance.

**Contribution:**

We present a case that can illuminate the utility of ultrasound in the diagnosis of pediatric neck masses. Additionally, we facilitate an important discussion on transoral versus transcervical approach for midline neck masses with the decision hinging on size, location, surgeon preference and comfort and consideration of cosmesis.

## Introduction

Worldwide, infants and children present yearly for evaluation of neck masses. Neck masses can be classified as benign, malignant, congenital, infectious, or inflammatory. Neck masses can be classified by location–most commonly midline versus lateral.^1^ The diagnostic dilemma resulting from the extensive list of potential diagnoses of a pediatric neck mass sparked a review of practice guidelines in recent years.^2^ Despite the analysis of pediatric neck masses in a few African countries,^3,4,5^ there remains a paucity of guidelines for pediatric neck masses in Zimbabwe. Here, we contribute to the literature on pediatric neck masses in Zimbabwe through a case presentation of a unique midline neck mass.

## Case presentation

ES was a 10-year-old female with no prior medical or surgical history who presented with her mother to a community pediatric hospital in Harare, Zimbabwe, for evaluation by the paediatric otolaryngology (ENT) clinic for an anterior neck mass. Her mother reported a 4-year history of a slowly enlarging mass, which began in the submental region as a small, non-painful lesion. They were offered antibiotic treatment for suspected abscess and referred to Harare Children’s Hospital. At that time, the patient’s mother was a widow without any income, and they were located 300 km from Harare. These challenges delayed their presentation for treatment for 3 years. Over this time, the mass continued to grow insidiously into her floor of mouth, displacing her tongue. This growth resulted in significant dysphagia and difficulty with speech perceptibility. Eventually, her mother saved enough money to pursue tertiary care.

**FIGURE 1:**
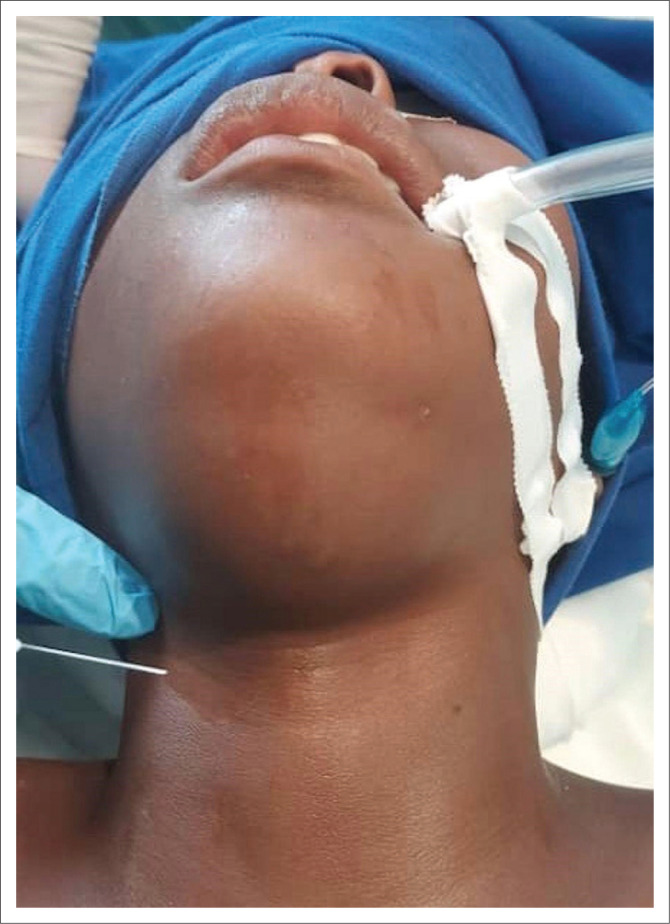
Pre-operative clinical photograph.

**FIGURE 2:**
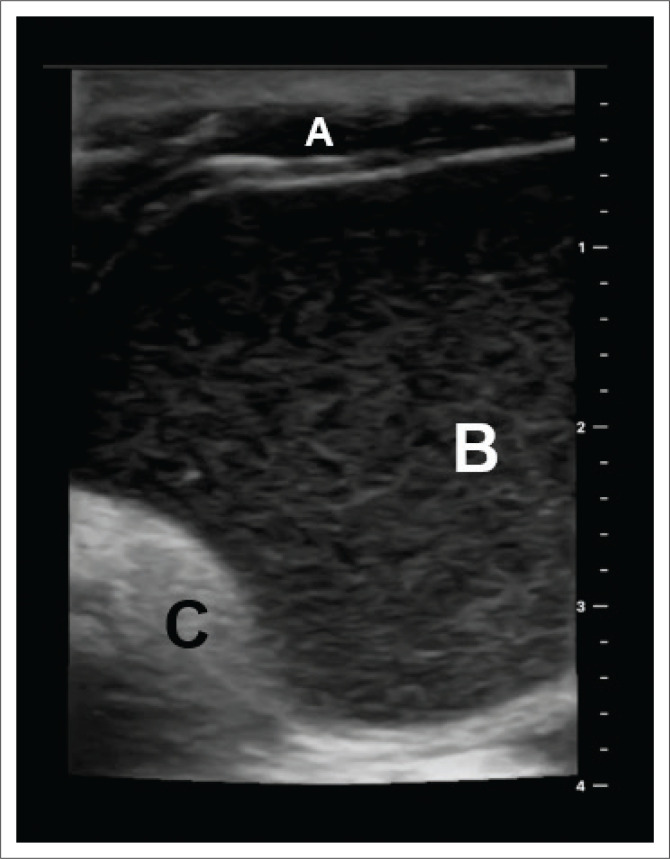
Pre-operative ultrasound imaging. The top of the image is superficial, whereas the bottom of the image is deep. The left of the image approaches the midline, while the right of the image is lateral. Letter ‘A’ marks the anterior belly of digastric. ‘B’ denotes the echogenic material found on the inside of the cyst. ‘C’ marks the posterior acoustic enhancement consistent with cystic structures along the mylohyoid.

On physical exam, her floor of the mouth was full and soft, and she had no appreciable dyspnoea; however, she experienced discomfort in a supine position (Figure 1). The patient did not have access to high-quality imaging such as computed tomography (CT) or magnetic resonance imaging (MRI); however, pre-operative ultrasound was donated (Figure 2) and was significant for a well-circumscribed midline neck mass. Our differential diagnosis was broad, including a plunging versus simple ranula, dermoid cyst, foregut duplication cyst, thyroglossal duct cyst or less likely abscess or malignancy.

**FIGURE 3:**
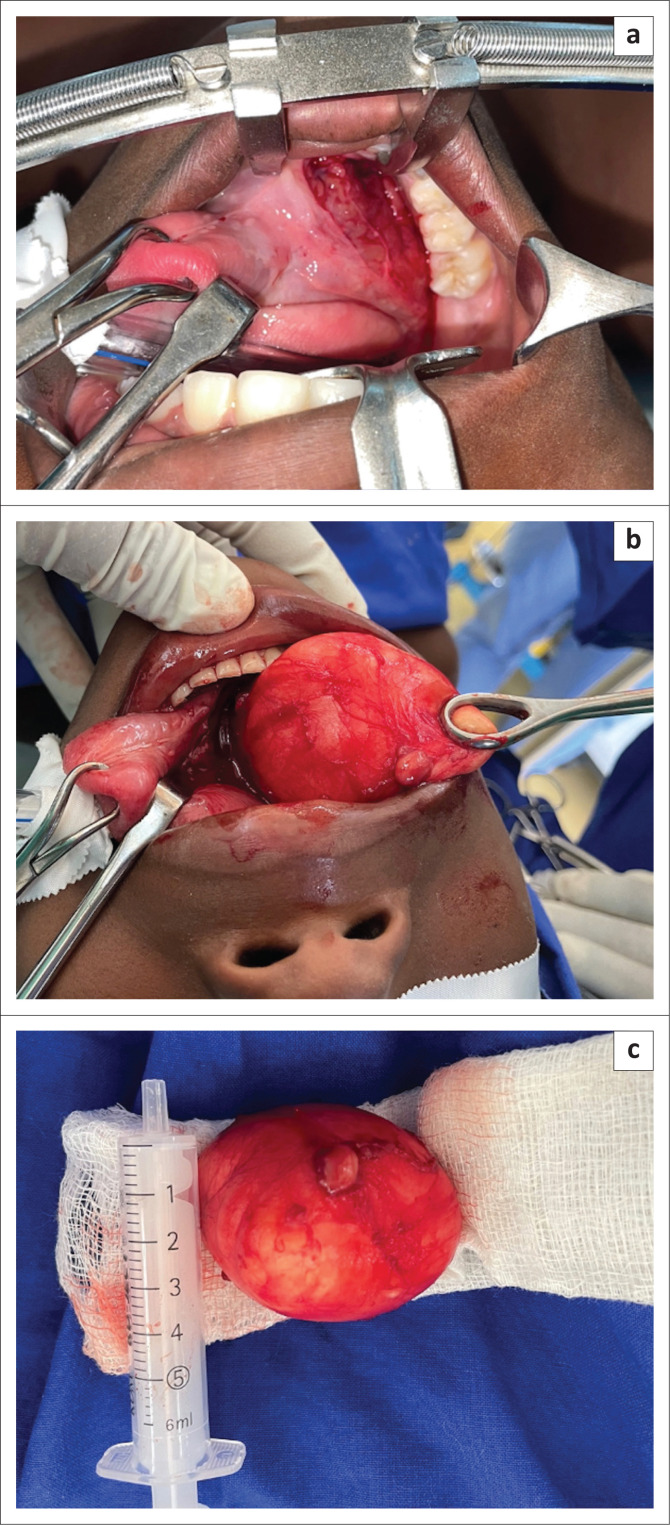
Intra-operative approach and specimen. (a) Intra-oral approach dissecting posteriorly and inferiorly to the sublingual gland. (b) Sublingual delivery of midline neck mass. (c) Cystic specimen.

After discussion with ES’s mother, surgical excision was elected via a transoral approach. Careful blunt dissection with a cold steel technique was used to deliver the mass through the floor of the mouth (Figure 3). The mass was sent for permanent pathology, which was significant for a keratin-containing cyst composed of epidermis with adnexal structures in the wall consistent with a dermoid. At 1-month post-operatively, the patient experienced no complications and had complete resolution of her functional symptoms and appearance.

## Discussion

The literature regarding pediatric neck masses in Zimbabwe is sparse. In this report, we detail the journey of a 10-year-old female from Harare, Zimbabwe, who presented with a progressively enlarging anterior neck mass. Here, pre-operative ultrasound was found to be extremely useful in facilitation of the diagnosis of a dermoid in a setting where it was otherwise difficult to obtain high-quality, more expensive imaging. We contribute this case to the growing literature on pediatric neck masses in low- and middle-income countries (LMIC) with our discussion of this large, dermoid neck mass. Dermoid cysts typically occur in children younger than six and most often in the head and neck region.^6^ Our case is similar to others presented in the literature^7,8,9^; however, we would like to use this case as an opportunity to highlight the utility of ultrasound in LMICs, especially in peripheral and rural hospitals in these countries. The differential diagnosis is broad for pediatric neck masses but can be narrowed by a thorough history, physical exam and imaging.^10,11,12,13^

Our patient’s story highlights a systemic barrier–access to timely and effective diagnostic imaging, which often extends beyond individuals’ financial limitations. These challenges highlight a broader, more systemic issue rather than the patient’s financial limitations. However, a simple yet effective imaging option in this scenario is the use of ultrasound. Because of its cost-effectiveness and ease of utility, ultrasound can be considered a first-line imaging modality, especially in LMICs.^14^ Dermoid cysts are defined on ultrasound by their well-demarcated nature with internal echogenicity and increased posterior acoustic enhancement.^14^ In this case, we appreciated the well-demarcated border of the mass just deep to the anterior belly of digastric filled with echogenic material and increased acoustic enhancement posteriorly that hid the mylohyoid muscle just deep to the mass (Figure 2). According to Stewart et al.^15^, the use of ultrasound is growing in LMICs as it is portable, cheap and can help with many diagnoses. We urge readers to consider using ultrasound in their algorithm for neck masses in Zimbabwe and other LMICs to help better define the mass and potentially save time and financial resources.

Moreover, the ability to characterise the mass is necessary for the next steps in management. In this case, surgical intervention was the only definitive management option; however, the discussion surrounding a transoral versus transcervical approach to the mass was less clear. One author^16^ reported an approach that included a combination of an extraoral and intraoral incision to be able to reach the mass from different angles. Pirgousis and Fernandes^17^ described a transcervical approach with the argument that this approach allowed decreased morbidity, shorter operative time, improved hemostasis, especially for larger masses that fill the submental space. Given our patient’s young age and lack of airway compromise with ample space to work in the floor of mouth, we ultimately decided on the transoral approach. Our decision-making was supported by Kim et al.^18^, who also reported that a transoral approach provides a safe and effective method provides a superior cosmetic outcome. We would recommend that the decision to perform a transoral versus transcervical approach should consider the expertise and comfort of the surgeon, size and location of the mass and aesthetic concerns of the patient.

Imaging can help better define the mass and guide steps in surgical management, but as with most head and neck pediatric masses, delays in care can make the management of any neck mass more difficult. There are three major delays described in the literature.^19^ A type 1 delay describes a delay in deciding to seek care, a type 2 delay is a delay in reaching the facility and a type 3 delay is a delay in receiving care at a healthcare facility. Our patient suffered from all three types of delays. This case underscores the need to address systemic delays in healthcare with a special focus on early and accessible imaging. Ultrasound is cost-effective and widely available. It also is instrumental to both surgical decision-making and planning.

## Conclusion

In summary, we present the case of a 10-year-old female who presented with a midline neck dermoid cyst. The significance of this case is the paucity of literature related to midline neck masses and their management in Zimbabwe and other LMICs. We hope to present a case that can illuminate the utility of ultrasound. Additionally, when considering the transoral versus transcervical approach for midline neck masses, we recommend that surgeons consider skill and experience, size, location of the mass and even cosmesis in their surgical planning. Lastly, we hope that providers consider the delays patients face in receiving care and work to improve access to timely surgical care and ultimately provide the best care for future patients.
